# Divergent morphological responses to millennia of climate change in two species of bats from Hall’s Cave, Texas, USA

**DOI:** 10.7717/peerj.10856

**Published:** 2021-03-15

**Authors:** Molly Moroz, Illiam S.C. Jackson, Daniel Ramirez, Melissa E. Kemp

**Affiliations:** Department of Integrative Biology, University of Texas at Austin, Austin, TX, United States of America

**Keywords:** Chiroptera, Climate change, Evolution, Mammalogy, Geometric morphometrics, Fossil, Paleobiology, Bats, Texas, Morphology

## Abstract

How species will respond to ongoing and future climate change is one of the most important questions facing biodiversity scientists today. The fossil record provides unparalleled insight into past ecological and evolutionary responses to climate change, but the resource remains virtually untapped for many organisms. We use geometric morphometrics and a 25,000 year fossil record to quantify changes in body size and mandible shape through time and across climate regimes for two bat species present in Quaternary paleontological deposits of central Texas: *Myotis velifer*, a bat distributed throughout the Southwestern US and Mexico that is still found in central Texas today, and *Eptesicus fuscus*, a bat widely distributed throughout North America that has been extirpated in central Texas. Because of ecogeographic rules like Bergmann’s rule, which posits that endotherms are larger in colder environments, we hypothesized that both species were larger during cooler time intervals. Additionally, we hypothesized that both species would show variation in dental morphology across the studied sequence as a response to climate change. While we found a decrease in centroid size–a proxy for ­­body size–through time for both species, we could not establish a clear relationship between centroid size and temperature alone. However, we did find that specimens from drier environments were significantly larger than those from wetter ones. Furthermore, we found significant dental shape variation between environments reflecting different temperature levels for both species. Yet only *M. velifer* exhibited significant variation between environments of varying precipitation levels. This result was surprising because present-day populations of *E. fuscus* are highly variable across both temperature and precipitation gradients. We determined that the morphological change experienced by *M. velifer* through time, and between warmer and cooler temperatures, was associated with the coronoid process, condylar process, and the mandibular symphysis. These parts play a pivotal role in bite force, so changes in these features might relate to changes in diet. We show that long-term datasets derived from fossil material provide invaluable insight not only into the validity of ecogeographic rules, but also into the adaptive capacities of extant taxa when faced with environmental changes. Our results highlight diverging responses to a variety of climate factors that are relevant to consider in biodiversity research given ongoing global change.

## Introduction

Climate change poses one of the most significant threats to the persistence of species in the Anthropocene. Changes in temperature and precipitation regimes have resulted in population declines, range shifts, extirpations, and extinctions across broad taxonomic groups of vertebrates; these effects are expected to intensify in coming centuries (Retallack, 2002; Davis, Shaw & Etterson, 2005; Parmesan, 2005; Solomon et al., 2009; Adams, 2010; Blois et al., 2013; Garcia et al., 2014; IPCC, 2014). Understanding the interplay of climate, species ecology, and phenotypic diversity is essential for predicting how species will be impacted by climate change. One phenotype that is significantly shaped by climate is morphology, as numerous studies illustrate (e.g., Bruzgul, Long & Hadly, 2005; McGuire, 2010; Yue et al., 2020). Morphological traits help organisms navigate their environments, and many organisms have evolved morphologies uniquely suited to environmental parameters such as trophic level (Freeman, 2000). Dental morphology is highly correlated to diet in mammals (Evans & Pineda-Munoz, 2018), and because climate determines resource availability (e.g., vegetation, prey type, and prey abundance), we expect that climate change will lead to morphological changes. Thus, data on the relationship between morphology and climate are critical to understanding how species will operate in changing environments.

Additionally, biogeographic patterns such as Bergmann’s rule, which posits that body size is inversely correlated with temperature, present a theoretical framework for how body size might vary under different climate regimes. Bergmann’s rule has found support in a multitude of studies looking at taxa over varying spatial scales, but its temporal longevity remains poorly understood (Meiri & Dayan, 2003; Gienapp et al., 2008; Teplitsky & Millien, 2014; Gohli & Voje, 2016). A recent evaluation of the relationship between body size and temperature in historical (centennial-scale) datasets found varying support for Bergmann’s rule (Gardner et al., 2011). Furthermore, a paucity of longer time series, such as millennial-scale datasets, make it difficult to experimentally test whether body size varies with climate.

Here, we use the fossil record to evaluate whether two temperate bat species (Mammalia: Chiroptera) underwent morphological changes that correspond to climate change. Bats are the second most speciose mammal order and provide important ecosystem services such as pollination, pest control, and seed dispersal around the world. However, climate change is likely to have many negative effects on bats by impacting their foraging, roosting and reproductive behaviors; increasing the prevalence of fungal diseases; and making portions of their current ranges uninhabitable (Sherwin, Montgomery & Lundy, 2013). Despite great potential and a present-day urgency to conserve bat biodiversity and ecosystem services, fossil bats are understudied relative to other mammalian orders due to sampling biases in the fossil record. To our knowledge, only a few studies have considered the long-term impacts of climate change on bat morphology, and most of these studies have focused solely on historical data; in two cases the bat populations evaluated were also experiencing increased urbanization, which makes it difficult to disentangle climate change’s impact from other global change phenomena (Tomassini et al., 2014).

Our work builds on one of the only millennial-scale studies of bat morphology through time: Toomey’s (1993) analyses of several bats from Hall’s Cave, Texas, United States. Hall’s Cave is one of North America’s most continuous faunal assemblages, spanning the last 20,000 years. We focus on two species: the cave myotis, *Myotis velifer,* and the big brown bat *Eptesicus fuscus. M. velifer* is distributed throughout Mexico and the southwestern United States, whereas *E. fuscus* is widely distributed throughout North America, the Caribbean, Central America, and the north-western region of South America. Of bat species known from Pleistocene fossil assemblages, *E. fuscus* is the most widely distributed (Kurta & Baker, 1990). Across their ranges, both species exhibit morphological variation, leading to an expectation that ancient populations would have adaptations for specific climatic regimes. Additionally, both species are opportunistic insectivores whose diets display seasonality and habitat variability (Harvey, Altenbach & Best , 2011). Toomey (1993) measured the lower tooth row length between the first through third molars and documented a significant downward trend in fossil size for *Myotis velifer* throughout the late Quaternary but found no trend in size change for *Eptesicus fuscus.* Interestingly, the two species have different fates that may be tied to their phenotypic responses: whereas *M. velifer* persists in Central Texas today, *E. fuscus* was extirpated at least 2,600 years before present (ybp; Toomey, 1993). Because climate fluctuations during this time interval were not unidirectional, it is possible that climate-correlated shifts in body size for *E. fuscus* were obfuscated by the Holocene climatic optimum, meaning that a comparison of traits across different climatic regimes might be more meaningful than an analysis across time. This is supported by the fact that presently, *E. fuscus* strongly abides by Bergmann’s rule (Burnett, 1983).  Furthermore, in the case of *M. velifer,* just as the overall size of bat cranial and dental elements changed through time, changes in morphology may have occurred that might not have been captured by linear measurements or qualitative observation, but which could have impacts on function (Zelditch, Swiderski & Sheets, 2012). Conversely, *E. fuscus* may have undergone morphological change in lieu of size change.

We reevaluate body size shifts in fossil *M. velifer* and *E. fuscus* by comparing dentary centroid size, a proxy for body size, across climate regimes and employ geometric morphometrics to quantify changes in dentary morphology of these taxa from Toomey’s original Hall’s Cave dataset, as well as three additional cave sites in Central Texas, collectively representing over 25,000 years of climate change. We hypothesized that *M. velifer*, already known to have exhibited changes in size through time in central Texas, would likewise change in dentary shape in response to climate change. Despite not having previously displayed a change in size through time, we expect that *E. fuscus* was larger during cooler time periods—consistent with Bergmann’s rule—and that the species will show variation in dentary morphology through the studied sequence as a response to climate change, in accordance with modern geographical variation across climate gradients of *E. fuscus. *

## Materials & Methods

### Geographical setting: the Edwards plateau

**Figure 1:**
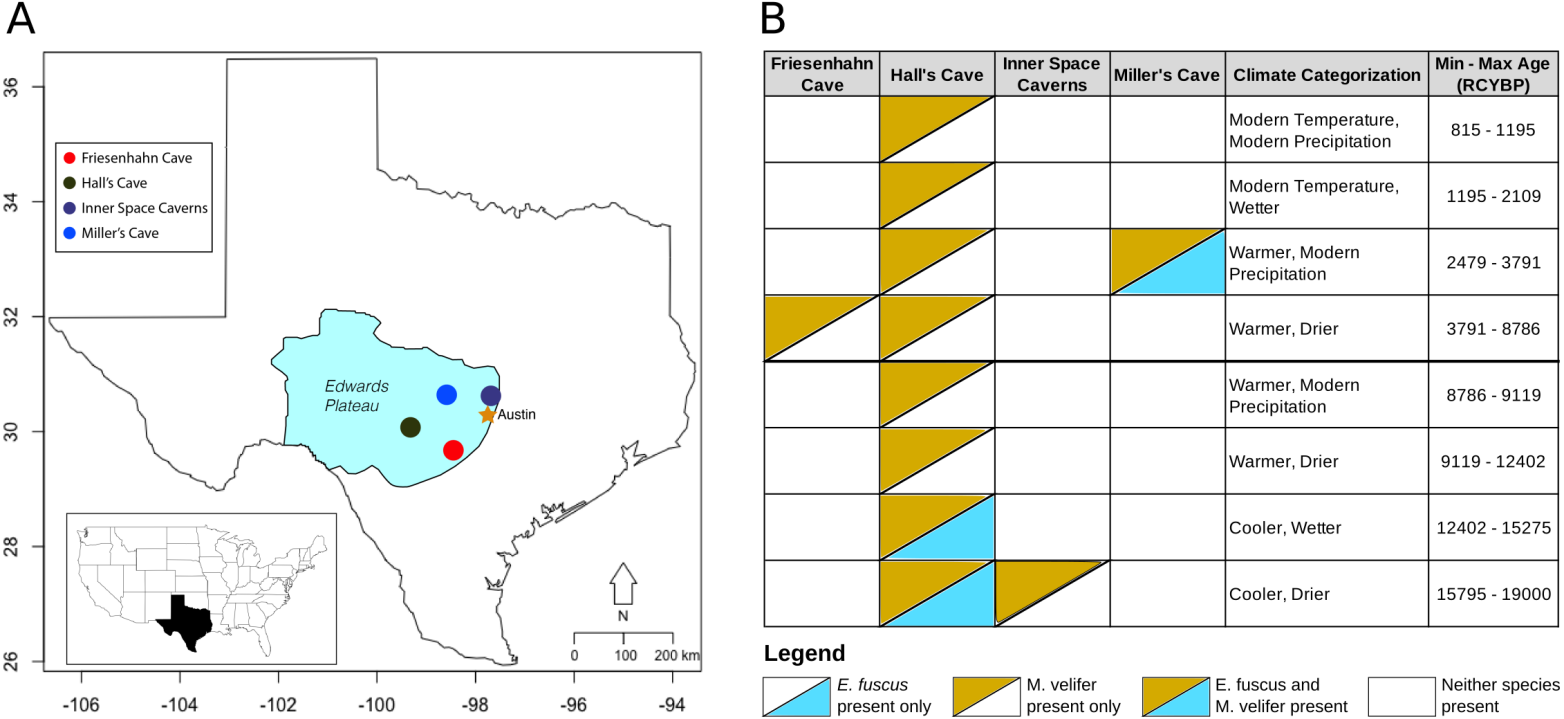
Map of the study region with climate categorizations and species presence. (A) Each of the fossil localities included in this study are caves occurring on the Edwards Plateau of central Texas. (B) The distribution of *E. fuscus* and *M. velifer* across the four cave sites, with the climate categorizations and specimen ages (in radiocarbon years before present, RCYBP) depicted. Climate categorizations follow (Cordova & Johnson, 2019).

All fossil dentaries of *Myotis velifer* and *Eptesicus fuscus* used in this study were excavated from caves in the Edwards Plateau of Central Texas (Fig. 1). Cretaceous marine deposits of limestone, sandstone, shales, and dolomite form the bedrock of the region. Dissolution of these soft sedimentary deposits resulted in the formation of numerous caves throughout the plateau. In the case of bats, which otherwise lack an extensive fossil record, fossiliferous cave deposits represent unique opportunities to study the paleoecology, evolution, and population dynamics of bats. We investigated the morphological change in dentaries of *M. velifer* and *E. fuscus* using fossils excavated from four cave sites on the Edwards Plateau, detailed below (Fig. 1). All fossil specimens are curated in the Vertebrate Paleontology Collections (VPC) at The University of Texas at Austin. A list of all specimens used in this study is included in the Supplemental Materials.

### Fossil localities

#### Friesenhahn cave

Friesenhahn Cave is located on the southeast edge of the Edwards Plateau in Bexar County, TX. It contains three distinct fossil-bearing temporal units dated to 17,000–19,000 RCYBP, 8,000–9,000 RCYBP, and <300 RCYBP, with *M. velifer* present in the intermediate unit representing the early Holocene (Toomey, 1993). *E. fuscus* is not present in this cave.

#### Hall’s Cave

About two-thirds of the fossils used in this study were collected in Hall’s Cave in Kerr County, TX. Hall’s Cave is famous for its long (>20,000 years), continuous sequence with abundant fossils representing the late Pleistocene-Holocene fauna of Central Texas and consequently has been the site of multiple studies of paleoenvironmental and faunal community change through time (Toomey, 1993; Toomey, Blum & Valastro, 1993; Smith et al., 2016). Fossils of *M. velifer* and *E. fuscus* were excavated from Hall’s cave over the course of several excavations starting in 1966 (Roth, 1972; Toomey, 1993). For this study, we used previously identified fossils spanning excavations from 1966 to those of Toomey (1993).

#### Inner space caverns

Formerly known as Laubach Cave, the Inner Space Caverns are located north of Austin, TX in Williamson County. Now a commercial cave, fauna from this site were originally described by Slaughter (1966) and Lundelius (1985). *M. velifer* fossils were found in one of the five fossil-bearing talus cones that make up the site: Laubach III. Laubach III was dated to 23,230 ±  490 RCYBP, representing the oldest fossils incorporated in this study (Toomey, 1993). *E. fuscus* were not recovered from this cave.

#### Miller cave

Miller Cave is located in Llano County, TX and contains two distinct fossil bearing units. The older of the two was dated to 7200 ±  300 RCYBP but contained neither *M. velifer* nor *E. fuscus* (Toomey, 1993). The younger unit was dated to 3008 ±  410 RCYBP and includes both *M. velifer* and *E. fuscus.*

### Data acquisition

#### Climate data

Historically, a variety of environmental proxies have been used to infer climate change in the Quaternary of Texas, including magnetic susceptibility, speleothems, and stable carbon isotopes, but climate estimations from these proxies can vary (Wong, Banner & Musgrove, 2015). We used climate inferences reported by Cordova & Johnson (2019), who deduced regional climatic trends for central Texas using changes in vegetation and faunal tolerances. The faunal tolerance data used by Cordova & Johnson (2019) come from Toomey (1993). This proxy relies on presence/absence data and knowledge of climatic tolerances for temperature and moisture sensitive small mammals present in Quaternary fossil assemblages of central Texas, including *Notiosorex crawfordi*, *Blarina brevicauda, Cryptotis parva, Tamias striatus,* and multiple *Sorex* species. As in Cordova & Johnson (2019), temperature is described as either warmer, cooler, or similar to the modern mean annual temperature, and precipitation is described as either wetter, drier, or similar to modern mean annual precipitation. The nearby city of Kerrville, TX has a mean annual temperature of 18 °C and receives 32 inches of precipitation per year. Different combinations of temperature categories occurred through time, so we compared size and shape data across temperature, precipitation, and combined temperature/precipitation groups.

#### Geometric morphometric data

We measured fossils from the Texas Vertebrate Paleontology Collection (TMM) and selected only complete, unbroken dentaries (*n* = 181). We used 29 specimens of *E. fuscus* and 152 specimens of *M. velifer.* We elected to use dentaries for our analyses because post-cranial skeletal elements of bats are rare in Quaternary deposits, and cranial elements like dentaries have been shown to correlate strongly with body size across mammals (Damuth et al., 1990). Museum identification numbers are included in the Supplemental Data. All specimens were previously identified to *M. velifer* or *E. fuscus* using documented morphological characters such as coronoid process shape and robustness of the ramus and by size; for this study, we assumed that all identifications were accurate (Toomey, 1993). *M. velifer* is significantly larger than any of the medium-sized *Myotis* species that have also been identified in Hall’s Cave and is thus much more easily differentiated than other species (Fitch, Shump & Shump, 1981; Toomey, 1993). By size, *E. fuscus* could only be misidentified as *Lasiurus cinereus,* but is easily distinguished by the shape of its coronoid process (Toomey, 1993). To maximize sample size, we disregarded the presence or absence of teeth because the number of teeth preserved in each dentary varied from a complete lack thereof to the presence of all ten teeth.

To quantify variation in dentary morphology through time, we photographed fossils in buccal view at 10X magnification using a Leica S9D stereo microscope with LASX software. Three photos were taken, and the two most focused and best illuminated photos were chosen for the geometric morphometrics analysis. We digitized the photos using TPSUtil and TPSDig following methods from Zelditch, Swiderski & Sheets (2012), Rohlf (2006). Landmarks were placed following conventions of previous studies using geometric morphometrics with bat dentaries (i.e., Nogueira, Peracchi & Monteiro, 2009; Sztencel-Jabłonka, Jones & Bogdanowicz, 2009; Jansky, Schubert & Wallace, 2016). However, many of our specimens manifested imperfections such that we could not use some of the intermediary type II landmarks employed by previous workers. For example, we could not comfortably identify the apex of the curvature formed between the posterior-most points of the angular and condylar processes, nor the apex of the curvature formed between the posterior-most point of the condylar process and the superior tip of the coronoid process. Instead, we chose to capture such variation with semi-landmarks, which are less susceptible to the impacts of putative imperfections. Furthermore, by disregarding tooth absence we naturally could not landmark teeth, nor could we use the occasionally abraded dentary rows as type I landmarks. Thus our methodological approach prioritized sample size over (fixed) landmark number.

**Figure 2:**
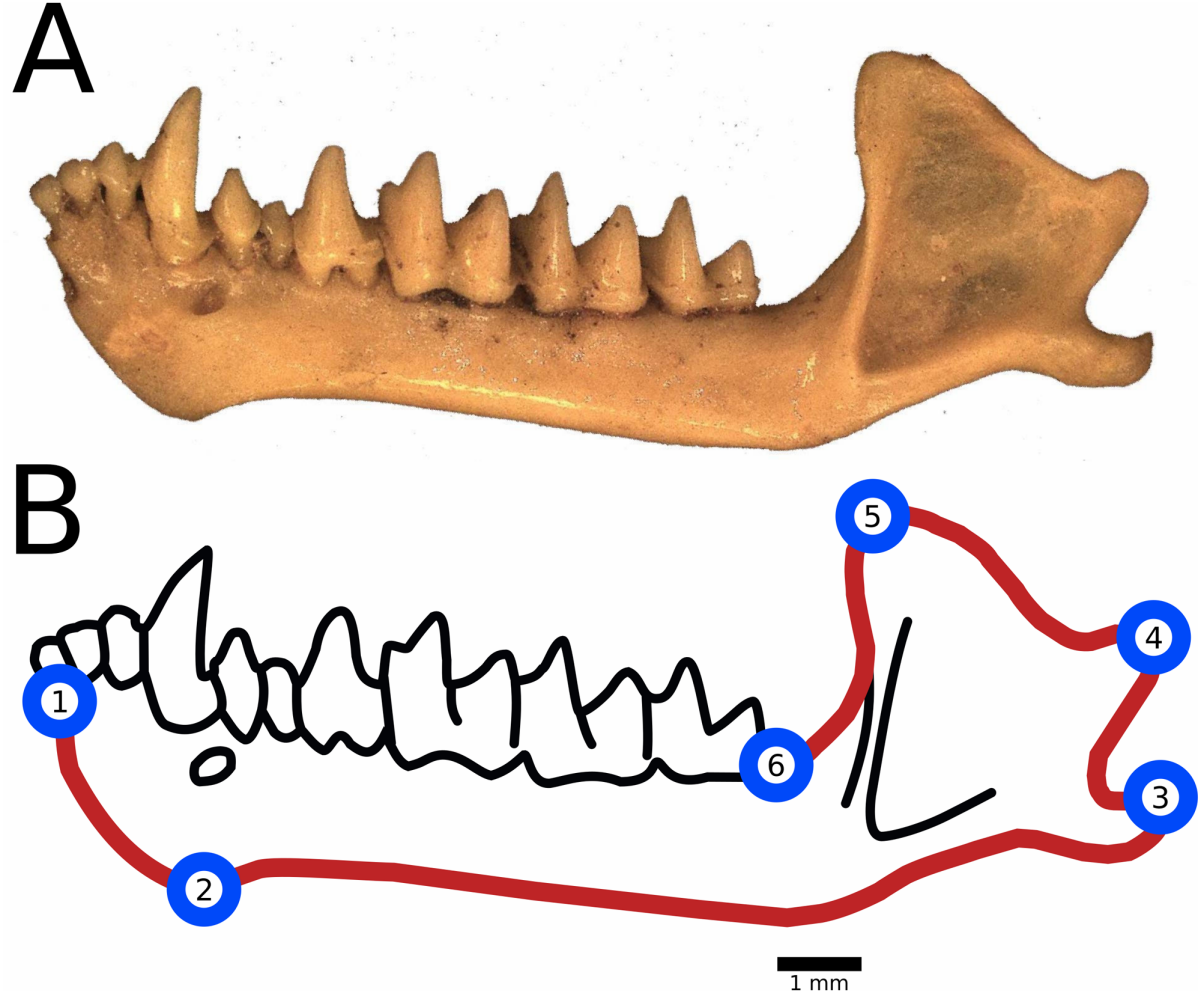
Landmark and semilandmark placement for geometric morphometrics. (A) An example of a fossil dentary of *M. velifer* collected from Hall’s Cave (TMM 41229-610). (B) Landmarks were placed at the following anatomical features: (1) Anterior-most point of the dentary, in the insertion of the inner lower incisors; (2) Posterior-most point of the mandibular symphysis; (3) Posterior-most point of the angular process; (4) Posterior-most point of the condylar process; (5) Superior tip of the coronoid process; (6) Posterior-most point of the last lower molar, at the level of insertion in the dentary bone. Semilandmark curves were used to trace outer edge of the mandible, symbolized here with a red line.

Landmark placement and anatomical descriptions of landmarks are included in Fig. 2. We placed semi-landmark curves tracing the outline of the dentary from lower molar (LM) 1 to LM 2, from LM 2 to LM 3 and so on. No semi-landmark curve connected LM 6 to LM 1 due to differences in preservation among the fossils resulting in non-biological variations in the dentaries through the region, such as breakage in the bone and mineral deposits. We calculated centroid size as the square root of the sum of squared distances of landmarks from the centroid of the digitized specimen and used this measure as a proxy for body size (Klingenberg, 2016).

All downstream statistical analyses were conducted in RStudio version 1.2.1335, using the additional packages “geomorph” version 3.1.2 (Adams & Otárola-Castillo, 2013) and “Morpho” version 2.8 (Schlager, 2017). For each species, we tested the association between age and size using a linear model (function lm in “stats” package), and investigated the relationship between centroid size and climate groups using pairwise t-tests between different environments (function pairwise.t.tests in “stats” package). For our shape analysis we conducted a generalized Procrustes analysis (GPA) on our landmark data (function gpagen in “geomorph” package) and tested for associations between the aligned Procrustes coordinates and our climate groups using permutation tests for group differences (function permudist in “Morpho” package). Next, we used Procrustes ANOVA with permutation (function procD.lm in “geomorph” package) to determine the relationship of aligned Procrustes coordinates relative size and age using the model [shape ∼size*age]. Finally, in order to visually examine putative morphological differences in those instances where significant variation was detected, we examined resultant principal components (PCs). For each PC from our GPA that described over 10% of the variation we tested for significant correlation with age and size using a linear model (function lm in “stats” package), and used permutation tests for group differences (function permudist in “Morpho” package) to investigate their individual relationships with climate groups, doing so only in instances where significant variation was detected by our preceding analysis using aligned Procrustes coordinates. Where relevant, we adjusted for multiple comparisons per Holm (1979). A presentation of our full suite of tests is available in the Supplemental Data.

## Results

### Centroid size

**Figure 3:**
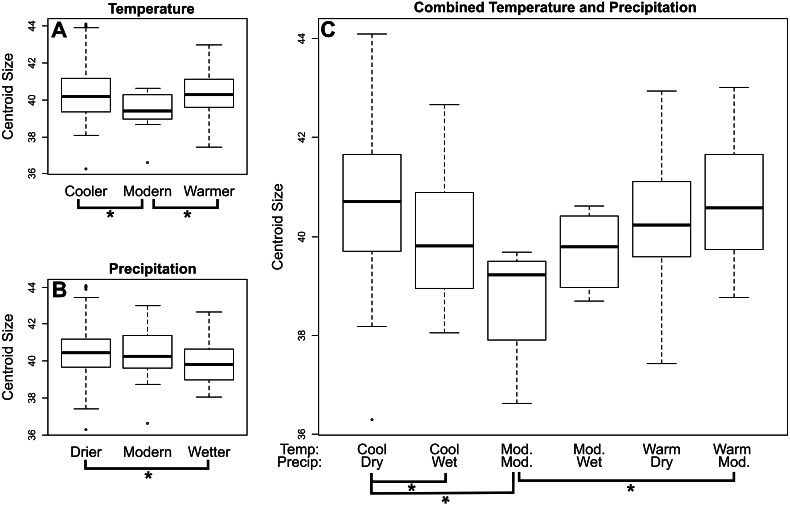
Mean centroid size of dentaries of *M. velifer* across temperature (A), precipitation (B), and combined climate (C) groups. Brackets with asterisks indicate significant differences in size between pairs (*p* value < 0.05). Tables including *p*-values for all pairwise comparisons are included in the Supplemental Data.

In *M. velifer,* we found a significant (*p* = 0.0478) trend of decreasing centroid size towards the present across all localities when considering the upper age estimate of our specimens and a highly indicative (*p* = 0.053) relationship of the same when considering the lower age estimate. We also compared centroid size across climate regimes (Fig. 3; Tables S1–S3); by calculating pairwise distances. We found that *M. velifer* specimens from environments of modern temperatures are significantly (*p* = 0.014) smaller than those from environments that were either warmer or cooler (Fig. 3A). Furthermore, *M. velifer* specimens associated with environments that were drier compared to modern precipitation levels were larger than specimens from environments that were wetter (*p* = 0.0025; Fig. 3B). We also measured pairwise differences for combined temperature and precipitation records (Fig. 3C; Table S3). We found that specimens from environments with both modern temperature and modern precipitation were significantly smaller than those from warmer environments with modern precipitation levels (*p* = 0.0421) and environments that were both cooler and drier (*p* = 0.0156). Furthermore, we found that specimens from cooler and drier environments were larger than those from cooler and wetter environments (*p* = 0.0014).

**Figure 4:**
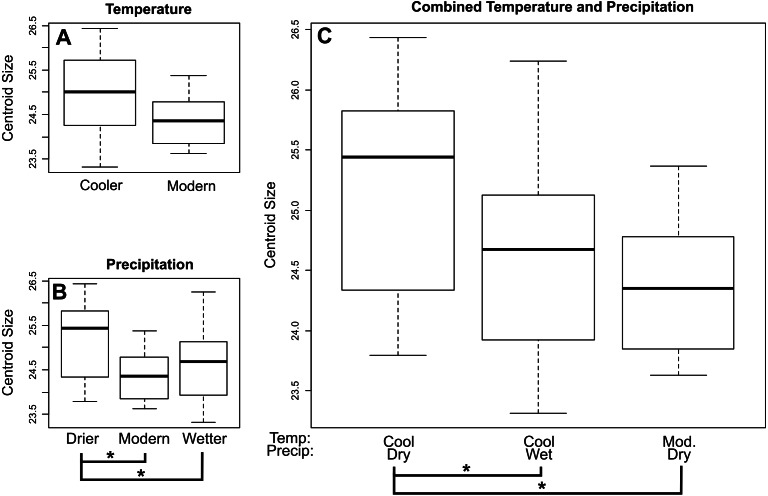
Mean centroid size of dentaries of *E. fuscus* across temperature (A), precipitation (B), and combined climate (C) groups. Brackets with asterisks indicate significant differences in size between pairs (*p* value < 0.05). Tables including *p*-values for all pairwise comparisons are included in the Supplemental Data.

For *E. fuscus* our analysis also recovered a decrease in centroid size through time towards the present when using both lower (*p* = 0.0199) and upper (*p* = 0.00724) age estimates. When comparing *E. fuscus* across climate regimes (Fig. 4; Tables S7–S9), we found no significant relationship between size and temperature, although we did detect a general trend of specimens from environments of modern temperature being smaller than those from drier environments (*p* = 0.072). We found that specimens from environments that were drier than modern-day environments were larger compared to specimens from both wetter environments (*p* = 0.029) and environments with modern levels of precipitation (*p* = 0.029; Fig. 4B). When considering combined temperature and precipitation groups (Fig. 4C; Table S9), we found that specimens from cooler and drier environments were significantly larger than specimens from environments that either shared temperature levels but were wetter (*p* = 0.029) as well as those that shared precipitation levels but were of modern temperature levels (*p* = 0.029).

### Shape data

Among *M. velifer,* using our model examining the impact of age and size on shape, we found that both centroid size and age, using the more conservative lower age estimate, significantly predicted shape differences (*p* = 0.01), but that their interaction did not do so (*p* = 0.32). When splitting the dentaries of *M. velifer* into climate groups, we found significant differences in shape (Tables S4–S6). Specifically, among temperature groups, we found that the dentaries of *M. velifer* from environments that were warmer than modern temperature levels differed in shape from environments that were cooler (*p* = 0.0003). Among precipitation groups, pairwise comparisons showed that *M. velifer* specimens from climates inferred to be wetter than the present differed in shape from specimens from drier than modern precipitation levels (*p* = 0.0024). When combining inferred precipitation and temperature records, we found that *M. velifer* from the cooler, drier interval that represent the earliest ∼14,000 years of our covered period are significantly different in shape compared to three other environments (Table S6): those that are cooler and wetter than modern (*p* = 0.0015), warmer and drier than modern (*p* = 0.0015), and finally environments that are warmer but share modern precipitation levels (*p* = 0.0156).

**Figure 5:**
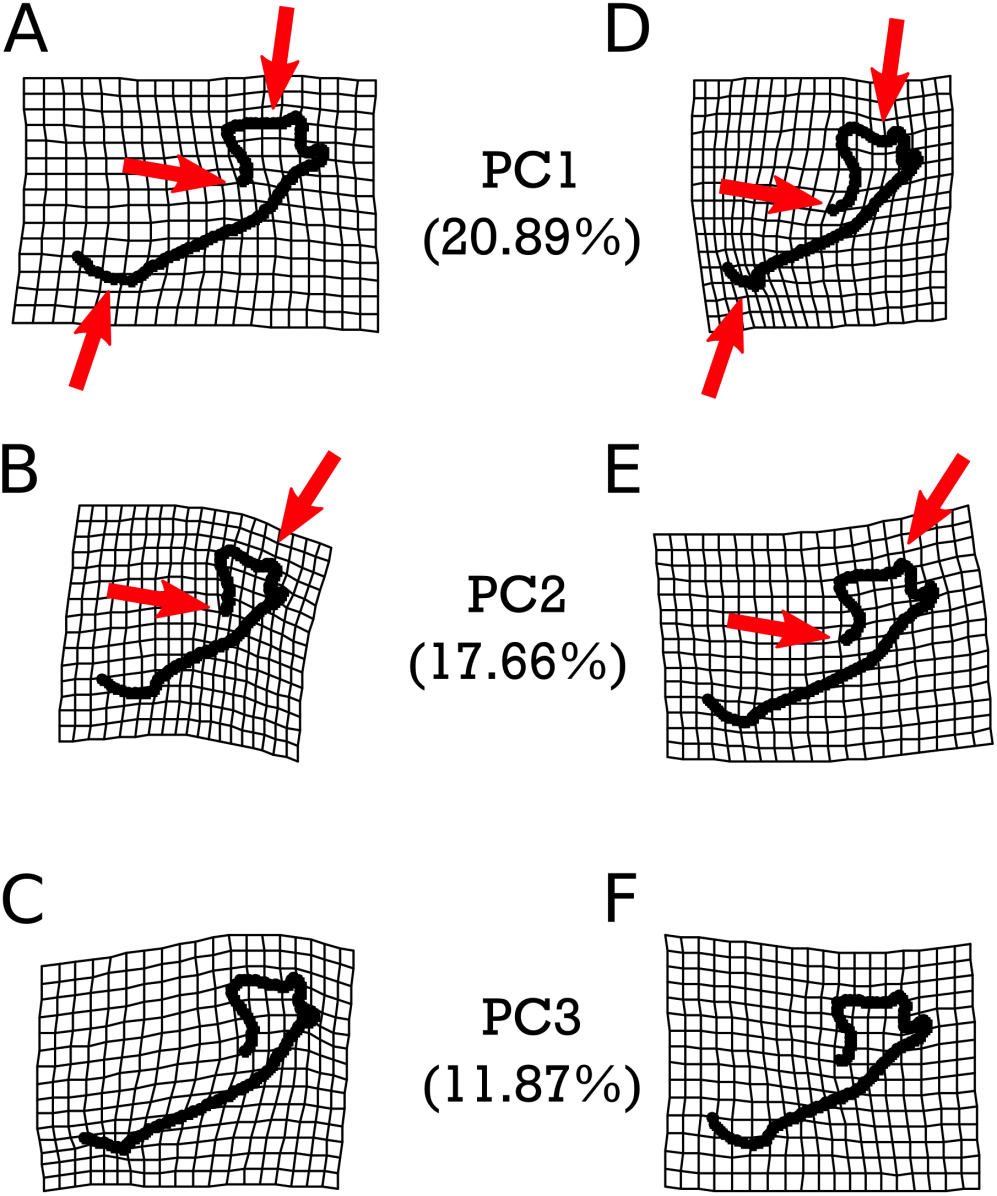
Warpgrids of the first three principal components of shape variation for *M. velifer*. Grids set to 2×  magnification. (A–C) indicate morphology of an individual exhibiting the minimum value for (A) PC1, (B) PC2, and (C) PC3. (D–F) demonstrate morphology of an individual exhibiting the maximum value for (D) PC1, (E) PC2, and (F) PC3. Red arrows indicate described anatomical features as major sources of variation for each PC: the mandibular symphysis (PC1), condylar process (PC1 & PC2), and coronoid process (PC1 & PC2).

Warp grids depicting the first three principal components (PCs) of shape variation in *M. velifer*, each of which describe more than 10% and cumulatively capture 50.42% of the total variation (Fig. S1 & Table S13), are presented in Fig. 5. We found that PC1 was significantly associated with the shape variation exhibited between specimens from cooler and drier environments relative modern levels and those that were warmer but drier (*p* = 0.0465). Apart from this instance, no further comparisons were significantly related to any of our climate groups (Figs. S2–S4 & Tables S15–Tables S23), nor were they predicted by centroid size (Table S24). However, PCs 1 and 2 (but not PC3) were also significantly predicted by specimen age when using both lower and upper age estimates (Table S14). These significant patterns are highlighted in Fig. 5. We found that PC1 was associated with the length of the mandibular symphysis, the flattening or curving of the condylar process, and a relative elongation or shortening of the dentary row. PC2 showed variation in the angle of the mandibular symphysis as well as compression or expansion of the condylar process.

**Figure 6:**
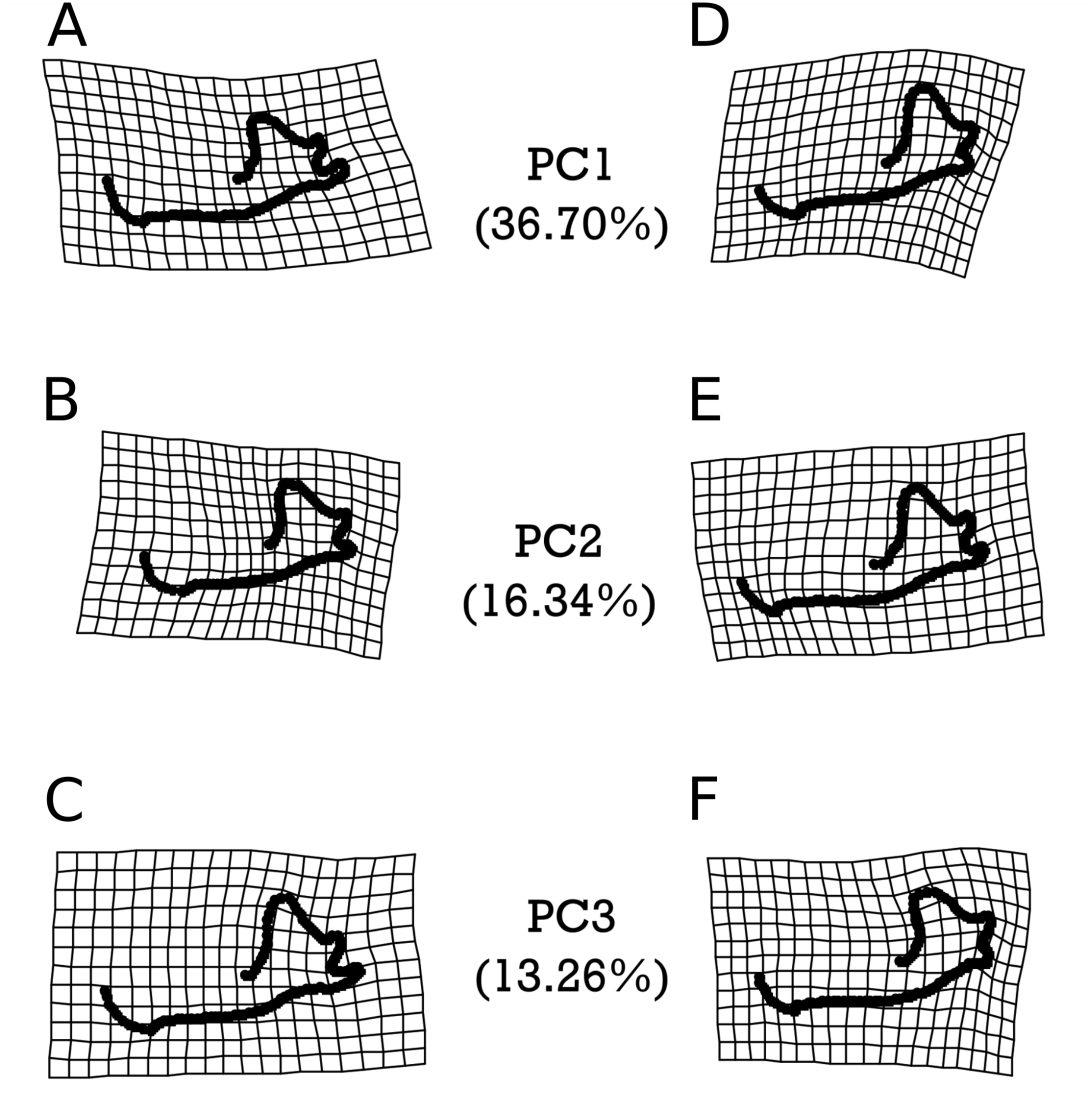
Warpgrids of the first 3 principal components of shape variation for *E. fuscus*. Grids set to 2×  magnification. (A–C) indicate morphology of an individual exhibiting the minimum value for (A) PC1, (B) PC2, and (C) PC3. (D–F) demonstrate morphology of an individual exhibiting the maximum value for (D) PC1, (E) PC2, and (F) PC3.

As with *M. velifer*, we also analyzed *E. fuscus* shape data using the same suite of tests. Our model investigating the impact of age and size on shape determined that neither of these factors on their own significantly predicted shape variation. However, their interaction was significantly (*p* = 0.022) correlated with a varying shape. This means that size is associated with shape changes, but that the response in shape for a given size depends on age; equally, age is associated with shape changes, but the response in shape for a given age depends upon its size. We did not find any significant correlations between shape and precipitation levels or combined climate groups (Tables S11–S12). However, specimens from environments inferred to be cooler than modern temperatures and those from environments equivalent to modern temperatures were significantly different (*p* = 0.0281). Figure 6 visualizes the morphological variation manifested along the first three principal components (PCs) of *E. fuscus*, each of which describe more than 10% and cumulatively capture 66.30% of the total variation (Fig. S1 & Table S13). We found that none of these PCs were individually significantly correlated with age (Table S25), temperature (Fig. S5 & Tables S26–S28), or size (Tables S29), despite each of these factors exhibiting significant trends in our preceding analysis of aligned Procrustes coordinates.

## Discussion

Our two focal taxa, *M. velifer* and *E. fuscus,* exhibit unique patterns of shape and size variation across climate categories over the past 25,000 years. While centroid size data in both *E. fuscus* and *M. velifer* indicate a decrease in body size over the past 25,000 years, we do not find that large body size is correlated with cooler time intervals as one would expect with Bergmann’s rule, under which mammals in colder environments are larger in body size, corresponding to an increase in surface area-to-volume ratio (Hayward, 1970). While *M. velifer* do exhibit a significantly larger size when comparing specimens from cooler environments to environments with modern temperature levels, and indeed the data indicate a similar pattern for *E. fuscus*, we do not find a significant size difference in *M. velifer* between specimens from cooler than modern environments compared to warmer than modern. For this reason, we are hesitant to conclude an association between colder environments and body size based on our data.

In modern North America, *E. fuscus* is more widely distributed than *M. velifer* and varies in size along temperature and precipitation gradients (Burnett, 1983). Thus, it was surprising that *E. fuscus* did not exhibit clear, directional shifts in body size across temperature, precipitation, or combined climate regimes. Our study is the first to test for Bergmann’s rule in bats using the fossil record and while we do not find support for Bergmann’s rule, we do uncover interesting relationships between precipitation and size for both *M. velifer* and *E. fuscus*, as both species are larger during time intervals that are drier than modern levels than those that are wetter. Our findings are more in line with James’ (1970) reformulation of Bergmann’s rule to account for the effect of desiccation on body size: James postulated that homeotherms would be larger in cool, drier environments and smaller in hot, humid environments. Still others have postulated that precipitation may correlate positively with body size because wet environments have higher resource availability (Burnett, 1983). Nevertheless, the relationship between body size and precipitation warrants more study to elucidate whether drier environments generate larger bats.

We also observed significant differences in dentary morphology across climate variables. Both *M. velifer* and *E. fuscus* differed in morphology across temperature groups. For the former, we found that specimens from warmer environments differed in shape compared to cooler ones, while for the latter we found a difference between the only groups represented in our data: environments of modern temperature levels and cooler ones. With regards to precipitation, we only found significant shape variation for *M. velifer*, which differed between drier-than-modern environments and wetter ones.

Taken together these results reveal that both *M. velifer* and *E. fuscus* experienced declines in body size throughout the late Quaternary of central Texas. While climatic variables could not be conclusively tied to this trend, we could show that temperature conditions influenced the morphology for both species, whereas precipitation levels only seemed to impact the mandibular morphology of *M. velifer* and not *E. fuscus*. Our results support those of Toomey (1993) wherein it was observed that tooth row length of *M. velifer* declined over time; we found evidence for this same trend alongside evidence that dentary shape also changed over time.

We show that most of the variation in morphology that was observed for *M. velifer* occurred in the mandibular symphysis and in the posterior end of the jaw, both of which play a significant role in the function of the jaw and teeth of the animal. Particularly, the shape and angle of the mandibular symphysis affects how lower incisor teeth are positioned in the jaw and how they interface with upper incisors, which affects both the types of insects caught by the bat and hunting success rate (Kunz, 1974). The posterior part of the dentary is engaged in a joint with the skull of the animal. The proportions and shape of the condylar process, angular process and coronoid process all play a role in the potential bite force exerted by the bat, and consequently, the hardness of insects that the bat can prey upon successfully (Nogueira, Peracchi & Monteiro, 2009; Hedrick & Dumont, 2018). Thus, variation in these morphological traits may be linked to changes in diet. While the late Quaternary insect fauna of central Texas remains poorly described, fossil data from West Texas and New Mexico indicate that insect diversity has shifted dramatically with climate and vegetation (Elias & Van Devender, 1992). Further research using methods such as stable isotope analysis could shed light on dietary shifts and the ecological implications of our work.

From these data, it is unclear if changes in morphology of the two focal taxa over different intervals were the result of dispersal, replacement of the population from neighboring colonies, or *in situ* adaptation within a single population. Modern *M. velifer* have been observed traveling up to 100 miles between caves for seasonal migration, which would mean that travel between any of the four caves studied here would be feasible (Tinkle & Patterson, 1965). Additionally, despite poor fossil records, many other caves on the Edwards Plateau could have housed populations of bats with varying rates of dispersal to the caves studied here. In the case of *E. fuscus,* modern ecological data indicate that the species utilizes caves only during cold intervals (Harvey, Altenbach & Best, 2011; Toomey, 1993) so as central Texas became warmer over the Quaternary, *E. fuscus* may have shifted away from using caves to roost. This may also explain the extirpation of *E. fuscus* from Central Texas which occurred as long ago as 2,500 ybp based on our dataset. While *E. fuscus* is much less abundant than *M. velifer* throughout the entire temporal sequence and is only found at two of our study sites, we do not think that these differences in sample size affect our results; if anything, they are indicative of differences in habitat use. For example, the lower abundances of fossils in more recent strata are consistent with the species transitioning to a few individuals using the cave rather than large colonies. Multiple scenarios of dispersal, extirpation, and migration may have played out, but the connectivity of populations and historic demographic events could be queried with ancient and modern DNA analyses.

## Conclusions

We show that long-term datasets derived from fossil materials provide invaluable insight not only into the validity of ecogeographic rules, but also into the adaptive capacities of extant taxa when faced with environmental changes. Both *M. velifer* and *E. fuscus* respond to varying temperatures and precipitation levels by altering their body size. Similarly, both exhibit shape variation as a consequence of temperature differences. However, only *M. velifer*, and not *E. fuscus*, experienced mandibular shape evolution in response to changing precipitation levels.

Whereas *M. velifer* persisted in a changing environment, *E. fuscus* has since become locally extirpated. While the causal relationship between these observations and the evolutionary patterns they represent will require further investigation to conclusively ascertain, these insights are only available through studying the fossil record, which represents a natural experiment in faunal responses to climate change over longer timescales than historic collections can provide. In recent decades, conservation has become an integral aspect of paleontology because of the discipline’s potential to shed light on past ecological and evolutionary responses in globally important species (e.g. Hunter, Jacobson & Webb, 1988; Dietl & Flessa, 2011; Pardi & Smith, 2012; Barnosky et al., 2017). While conservation efforts often prioritize range-restricted species over widespread taxa (Barnosky et al., 2017) our data indicate that the range-restricted *Myotis velifer* may be able to adapt to climate change. Although we report on a local extirpation of a widespread species, we cannot conclude from our data alone that the extirpation resulted from an inability to adapt morphologically; the population may have migrated to a more hospitable region. Yet it is crucial that we study populations of both widespread and range-restricted species and decipher how each species responds to climate change if we want to ensure the survival of populations”—and ultimately, species”—in the face of modern, rapid climate change.

##  Supplemental Information

10.7717/peerj.10856/supp-1 Supplemental Information 1 Supplemental Data Appendix

10.7717/peerj.10856/supp-2 Supplemental Information 2 *Myotis velifer* raw geometric morphometric data

10.7717/peerj.10856/supp-3 Supplemental Information 3 *Eptesicus fuscus* raw geometric morphometric data

## Additional Information and Declarations

### Data Availability

The following information was supplied regarding data availability: Data, including specimen accession numbers and raw shape data, are available in the Supplementary Files. All fossil specimens are curated in the Vertebrate Paleontology Collections (TMM) at The University of Texas at Austin.
