# Supplementary material for: Divergent morphological responses to millennia of climate change in two species of bats from Hall’s Cave, Texas, USA
Source: PeerJ. 2021 Mar 15;9:e10856. doi: 10.7717/peerj.10856 (PMC7971077; doi:10.7717/peerj.10856)
Supplement: Supplemental Information 1 [file peerj-09-10856-s001.docx]

List of specimens

| **Species** | **Specimen** | **Locality** |  |  |
| --- | --- | --- | --- | --- |
| E. fuscus | 40540-003 | Miller Cave | |  |
| E. fuscus | 40540-004 | Miller Cave | |  |
| E. fuscus | 40540-005 | Miller Cave | |  |
| E. fuscus | 40540-007 | Miller Cave | |  |
| M. velifer | 40540-132 | Miller Cave | |  |
| M. velifer | 40540-133 | Miller Cave | |  |
| M. velifer | 40540-202-2 | Miller Cave | |  |
| M. velifer | 41229-10043 | Hall’s cave |  |  |
| M. velifer | 41229-10046 | Hall’s cave |  |  |
| M. velifer | 41229-10048 | Hall’s cave |  |  |
| M. velifer | 41229-1038 | Hall’s cave |  |  |
| M. velifer | 41229-10477 | Hall’s cave |  |  |
| M. velifer | 41229-10533 | Hall’s cave |  |  |
| M. velifer | 41229-10534 | Hall’s cave |  |  |
| M. velifer | 41229-10888 | Hall’s cave |  |  |
| M. velifer | 41229-10889 | Hall’s cave |  |  |
| M. velifer | 41229-10950 | Hall’s cave |  |  |
| M. velifer | 41229-11099 | Hall’s cave |  |  |
| M. velifer | 41229-11174 | Hall’s cave |  |  |
| M. velifer | 41229-11285 | Hall’s cave |  |  |
| E. fuscus | 41229-11303 | Hall’s cave |  |  |
| M. velifer | 41229-11306 | Hall’s cave |  |  |
| M. velifer | 41229-11308 | Hall’s cave |  |  |
| M. velifer | 41229-11333 | Hall’s cave |  |  |
| M. velifer | 41229-11334 | Hall’s cave |  |  |
| M. velifer | 41229-11342 | Hall’s cave |  |  |
| M. velifer | 41229-11408 | Hall’s cave |  |  |
| M. velifer | 41229-1154 | Hall’s cave |  |  |
| M. velifer | 41229-11548 | Hall’s cave |  |  |
| M. velifer | 41229-11613 | Hall’s cave |  |  |
| M. velifer | 41229-11650 | Hall’s cave |  |  |
| M. velifer | 41229-1172 | Hall’s cave |  |  |
| M. velifer | 41229-11826 | Hall’s cave |  |  |
| M. velifer | 41229-11827 | Hall’s cave |  |  |
| M. velifer | 41229-11860 | Hall’s cave |  |  |
| M. velifer | 41229-11861 | Hall’s cave |  |  |
| M. velifer | 41229-11862 | Hall’s cave |  |  |
| M. velifer | 41229-11870 | Hall’s cave |  |  |
| M. velifer | 41229-11873 | Hall’s cave |  |  |
| M. velifer | 41229-11879 | Hall’s cave |  |  |
| M. velifer | 41229-11880 | Hall’s cave |  |  |
| E. fuscus | 41229-1191 | Hall’s cave |  |  |
| M. velifer | 41229-1197 | Hall’s cave |  |  |
| M. velifer | 41229-1199 | Hall’s cave |  |  |
| M. velifer | 41229-1203 | Hall’s cave |  |  |
| M. velifer | 41229-1228 | Hall’s cave |  |  |
| M. velifer | 41229-131 | Hall’s cave |  |  |
| E. fuscus | 41229-1739 | Hall’s cave |  |  |
| M. velifer | 41229-1753 | Hall’s cave |  |  |
| E. fuscus | 41229-1760 | Hall’s cave |  |  |
| M. velifer | 41229-1817 | Hall’s cave |  |  |
| M. velifer | 41229-2520 | Hall’s cave |  |  |
| E. fuscus | 41229-2685 | Hall’s cave |  |  |
| E. fuscus | 41229-2690 | Hall’s cave |  |  |
| E. fuscus | 41229-2717 | Hall’s cave |  |  |
| M. velifer | 41229-2967 | Hall’s cave |  |  |
| E. fuscus | 41229-3892 | Hall’s cave |  |  |
| E. fuscus | 41229-3894 | Hall’s cave |  |  |
| M. velifer | 41229-4051 | Hall’s cave |  |  |
| M. velifer | 41229-4122 | Hall’s cave |  |  |
| M. velifer | 41229-4123 | Hall’s cave |  |  |
| M. velifer | 41229-4564 | Hall’s cave |  |  |
| M. velifer | 41229-4810 | Hall’s cave |  |  |
| M. velifer | 41229-4856 | Hall’s cave |  |  |
| M. velifer | 41229-5450 | Hall’s cave |  |  |
| M. velifer | 41229-5462 | Hall’s cave |  |  |
| M. velifer | 41229-5471 | Hall’s cave |  |  |
| M. velifer | 41229-5496 | Hall’s cave |  |  |
| M. velifer | 41229-550 | Hall’s cave |  |  |
| M. velifer | 41229-566 | Hall’s cave |  |  |
| M. velifer | 41229-5733 | Hall’s cave |  |  |
| M. velifer | 41229-575 | Hall’s cave |  |  |
| M. velifer | 41229-5839 | Hall’s cave |  |  |
| E. fuscus | 41229-589 | Hall’s cave |  |  |
| E. fuscus | 41229-6012 | Hall’s cave |  |  |
| E. fuscus | 41229-6013 | Hall’s cave |  |  |
| E. fuscus | 41229-6014 | Hall’s cave |  |  |
| E. fuscus | 41229-6015 | Hall’s cave |  |  |
| E. fuscus | 41229-6017 | Hall’s cave |  |  |
| E. fuscus | 41229-6018 | Hall’s cave |  |  |
| E. fuscus | 41229-6028 | Hall’s cave |  |  |
| E. fuscus | 41229-6029 | Hall’s cave |  |  |
| E. fuscus | 41229-6030 | Hall’s cave |  |  |
| E. fuscus | 41229-605 | Hall’s cave |  |  |
| E. fuscus | 41229-607 | Hall’s cave |  |  |
| E. fuscus | 41229-6083 | Hall’s cave |  |  |
| M. velifer | 41229-610 | Hall’s cave |  |  |
| M. velifer | 41229-611 | Hall’s cave |  |  |
| M. velifer | 41229-6139 | Hall’s cave |  |  |
| M. velifer | 41229-614 | Hall’s cave |  |  |
| M. velifer | 41229-6184 | Hall’s cave |  |  |
| M. velifer | 41229-6187 | Hall’s cave |  |  |
| M. velifer | 41229-6189 | Hall’s cave |  |  |
| M. velifer | 41229-6226 | Hall’s cave |  |  |
| M. velifer | 41229-6230 | Hall’s cave |  |  |
| M. velifer | 41229-6233 | Hall’s cave |  |  |
| M. velifer | 41229-6234 | Hall’s cave |  |  |
| M. velifer | 41229-6311 | Hall’s cave |  |  |
| E. fuscus | 41229-632 | Hall’s cave |  |  |
| M. velifer | 41229-6564 | Hall’s cave |  |  |
| M. velifer | 41229-6565 | Hall’s cave |  |  |
| M. velifer | 41229-6566 | Hall’s cave |  |  |
| M. velifer | 41229-6567 | Hall’s cave |  |  |
| M. velifer | 41229-6568 | Hall’s cave |  |  |
| M. velifer | 41229-6570 | Hall’s cave |  |  |
| M. velifer | 41229-6571 | Hall’s cave |  |  |
| M. velifer | 41229-6572 | Hall’s cave |  |  |
| M. velifer | 41229-6573 | Hall’s cave |  |  |
| M. velifer | 41229-6638 | Hall’s cave |  |  |
| M. velifer | 41229-6660 | Hall’s cave |  |  |
| M. velifer | 41229-6663 | Hall’s cave |  |  |
| M. velifer | 41229-6677 | Hall’s cave |  |  |
| M. velifer | 41229-6681 | Hall’s cave |  |  |
| M. velifer | 41229-6713 | Hall’s cave |  |  |
| M. velifer | 41229-6714 | Hall’s cave |  |  |
| M. velifer | 41229-6717 | Hall’s cave |  |  |
| M. velifer | 41229-6874 | Hall’s cave |  |  |
| M. velifer | 41229-6974 | Hall’s cave |  |  |
| M. velifer | 41229-7072 | Hall’s cave |  |  |
| M. velifer | 41229-7080 | Hall’s cave |  |  |
| M. velifer | 41229-7081 | Hall’s cave |  |  |
| M. velifer | 41229-7136 | Hall’s cave |  |  |
| M. velifer | 41229-7137 | Hall’s cave |  |  |
| M. velifer | 41229-7142 | Hall’s cave |  |  |
| M. velifer | 41229-768 | Hall’s cave |  |  |
| M. velifer | 41229-789 | Hall’s cave |  |  |
| M. velifer | 41229-793 | Hall’s cave |  |  |
| E. fuscus | 41229-872 | Hall’s cave |  |  |
| M. velifer | 41229-8931 | Hall’s cave |  |  |
| M. velifer | 41229-8933 | Hall’s cave |  |  |
| M. velifer | 41229-8934 | Hall’s cave |  |  |
| E. fuscus | 41229-897 | Hall’s cave |  |  |
| M. velifer | 41229-899 | Hall’s cave |  |  |
| M. velifer | 41229-942 | Hall’s cave |  |  |
| M. velifer | 41229-9854 | Hall’s cave |  |  |
| M. velifer | 41229-9855 | Hall’s cave |  |  |
| M. velifer | 41229-9875 | Hall’s cave |  |  |
| M. velifer | 41229-9879 | Hall’s cave |  |  |
| M. velifer | 41229-9910 | Hall’s cave |  |  |
| M. velifer | 41229-9924 | Hall’s cave |  |  |
| M. velifer | 41229-9925 | Hall’s cave |  |  |
| M. velifer | 41229-9926 | Hall’s cave |  |  |
| M. velifer | 41229-9928 | Hall’s cave |  |  |
| M. velifer | 41229-9929 | Hall’s cave |  |  |
| M. velifer | 41229-9930 | Hall’s cave |  |  |
| M. velifer | 41229-9931 | Hall’s cave |  |  |
| M. velifer | 41343-193 | Inner Space Caverns | | |
| M. velifer | 41343-194 | Inner Space Caverns | | |
| M. velifer | 41343-195 | Inner Space Caverns | | |
| M. velifer | 41343-196 | Inner Space Caverns | | |
| M. velifer | 41343-197 | Inner Space Caverns | | |
| M. velifer | 41343-198 | Inner Space Caverns | | |
| M. velifer | 41343-199 | Inner Space Caverns | | |
| M. velifer | 41343-201 | Inner Space Caverns | | |
| M. velifer | 41343-202 | Inner Space Caverns | | |
| M. velifer | 41343-206 | Inner Space Caverns | | |
| M. velifer | 41343-207 | Inner Space Caverns | | |
| M. velifer | 41343-208 | Inner Space Caverns | | |
| M. velifer | 41343-209 | Inner Space Caverns | | |
| M. velifer | 41343-210 | Inner Space Caverns | | |
| M. velifer | 41343-211 | Inner Space Caverns | | |
| M. velifer | 41343-212 | Inner Space Caverns | | |
| M. velifer | 41343-213 | Inner Space Caverns | | |
| M. velifer | 41343-215 | Inner Space Caverns | | |
| M. velifer | 41343-219 | Inner Space Caverns | | |
| M. velifer | 41343-220 | Inner Space Caverns | | |
| M. velifer | 41343-222 | Inner Space Caverns | | |
| M. velifer | 41343-223 | Inner Space Caverns | | |
| M. velifer | 41343-224 | Inner Space Caverns | | |
| M. velifer | 41343-225 | Inner Space Caverns | | |
| M. velifer | 41343-226 | Inner Space Caverns | | |
| M. velifer | 41343-227 | Inner Space Caverns | | |
| M. velifer | 41343-228 | Inner Space Caverns | | |
| M. velifer | 41343-229 | Inner Space Caverns | | |
| M. velifer | 41343-230 | Inner Space Caverns | | |
| M. velifer | 933-3997 | Friesenhahn Cave | |  |
| M. velifer | 933-3999A | Friesenhahn Cave | |  |
| M. velifer | 933-4001A | Friesenhahn Cave | |  |
| M. velifer | 933-4001C | Friesenhahn Cave | |  |
| M. velifer | 933-4002 | Friesenhahn Cave | |  |
| M. velifer | 933-5003 | Friesenhahn Cave | |  |

*M. velifer*

Shape - Centroid Size ~ Minimum Age

**✔** *M. velifer* **shape** **is** significantly predicted by **centroid size**, when tested using a Procrustes ANOVA with permutation (procD.lm function in geomorph). **p=0.004**

**✔** *M. velifer* **shape** **is** significantly predicted by **minimum age** per specimen, when tested using a Procrustes ANOVA with permutation (procD.lm function in geomorph). **p=0.001**

**X** *M. velifer* **shape** **is not** significantly predicted by a combination of **centroid size** and **minimum age**, when tested using a Procrustes ANOVA with permutation (procD.lm function in geomorph package). p=0.228

Centroid Size - Climate

**✔** *M. velifer* **centroid size** **does** significantly vary across different temperature regimes, when tested using Pairwise T-Tests with correction for multiple testing (pairwise.t.test function in stats package). Specimens from environments with modern temperature levels are smaller than specimens from both cooler and warmer environments, which are indistinguishable relative to each other.

**Supplementary Table 1: *M. velifer* centroid size by temperature**

| p-values | **Cool** | **Modern** |
| --- | --- | --- |
| **Modern** | **0.014** | - |
| **Warm** | 0.844 | **0.014** |

**✔** *M. velifer* **centroid size** **does** significantly vary across different precipitation regimes, when tested using Pairwise T-Tests with correction for multiple testing (pairwise.t.test function in stats package). Specimens from dry environments are larger than specimens from wet environments.

**Supplementary Table 2: *M. velifer* centroid size by precipitation**

| p-values | **Dry** | **Modern** |
| --- | --- | --- |
| **Modern** | 0.7476 | - |
| **Wet** | **0.0025** | 0.1019 |

**✔** *M. velifer* **centroid size** **does** significantly vary across different climatic regimes, when tested using Pairwise T-Tests with correction for multiple testing (pairwise.t.test function in stats package).

The directionality of the difference is given by comparing **row** by **column**. E.g. **Cool, Wet** specimens are significantly **smaller** than **Cool, Dry** specimens.

**Supplementary Table 3: *M. velifer* centroid size by climate**

| p-values | **Cool, Dry** | **Cool, Wet** | **Modern Temp., Modern Precip.** | **Modern Temp., Wet** | **Warm, Dry** |
| --- | --- | --- | --- | --- | --- |
| **Cool, Wet** | **0.0014 (smaller)** | - | - | - | - |
| **Modern Temp., Modern Precip.** | **0.0156**  **(smaller)** | 0.3661 | - | - | - |
| **Modern Temp., Wet** | 0.0981 | 1.0000 | 0.7236 | - | - |
| **Warm, Dry** | 0.1106 | 0.3661 | 0.1106 | 0.5796 | - |
| **Warm, Modern. Precip.** | 1.0000 | 0.1106 | **0.0421**  **(larger)** | 0.2562 | 0.7236 |

Shape - Climate

**✔** *M. velifer* **shape** **does** significantly vary across different temperature regimes, when tested using Permutation Testing For Group Differences with correction for multiple testing (permudist function in Morpho package). Specimens from environments that are warmer than modern levels are significantly different in terms of shape to specimens from environments that are cooler than modern levels.

**Supplementary Table 4: *M. velifer* shape by temperature**

| p-values | **Cool** | **Modern** |
| --- | --- | --- |
| **Modern** | 0.1762 | - |
| **Warm** | **0.0003** | 0.3179 |

**✔** *M. velifer* **shape** **does** significantly vary across different precipitation regimes, when tested using Permutation Testing For Group Differences with correction for multiple testing (permudist function in Morpho package). Specimens from dry and wet environments differ from each other but no other group differences are significant.

**Supplementary Table 5: *M. velifer* shape by precipitation**

| p-values | **Dry** | **Modern** |
| --- | --- | --- |
| **Modern** | 0.4514 | - |
| **Wet** | **0.0024** | 0.4514 |

**✔** *M. velifer* **shape**  **does** significantly vary across different climatic regimes, when tested using Permutation Testing For Group Differences with correction for multiple testing (permudist function in Morpho package). Only cool dry environments differed from other environments.

**Supplementary Table 6: *M. velifer* shape by climate**

| p-values | **Cool, Dry** | **Cool, Wet** | **Modern Temp., Modern Precip.** | **Modern Temp., Wet** | **Warm, Dry** |
| --- | --- | --- | --- | --- | --- |
| **Cool, Wet** | **0.0015** | - | - | - | - |
| **Modern Temp., Modern Precip.** | 0.5400 | 1.0000 | - | - | - |
| **Modern Temp., Wet** | 0.1164 | 1.0000 | 1.0000 | - | - |
| **Warm, Dry** | **0.0015** | 0.2024 | 1.0000 | 1.0000 | - |
| **Warm, Modern. Precip.** | **0.0156** | 1.0000 | 1.0000 | 1.0000 | 1.0000 |

Centroid Size - Age

Each specimen is associated with an **Upper** and **Lower Age Estimate**. Using a Linear Model (lm function in stats package):

**X** *M. velifer* **Lower Age Estimate** **does not** significantly predict **centroid size**. p=0.053

**✔** *M. velifer* **Upper Age Estimate** **does** significantly predict **centroid size**. **p=0.0478**

**Older** specimens have a **larger** Centroid Size.

*E. fuscus*

Shape - Centroid Size ~ Minimum Age

**X** *E. fuscus* **shape** **is not** significantly predicted by **centroid size**, when tested using a Procrustes ANOVA with permutation (procD.lm function in geomorph). p=0.092

**X** *E. fuscus* **shape** **is not** significantly predicted by **minimum age** per specimen, when tested using a Procrustes ANOVA with permutation (procD.lm function in geomorph). p=0.129

**✔** *E. fuscus* **shape** **is** significantly predicted by a combination of **centroid size** and **minimum age**, when tested using a Procrustes ANOVA with permutation (procD.lm function in geomorph package). **p=0.022**

Centroid Size - Climate

**X** *E. fuscus* **centroid size** **does not** significantly vary across different temperature regimes, when tested using Pairwise T-Tests with correction for multiple testing (not applicable in this specific instance in any case; pairwise.t.test function in stats package).

**Supplementary Table 1: *E. fuscus* centroid size by temperature**

| p-values | **Modern** |
| --- | --- |
| **Cool** | 0.072 |

**✔** *E. fuscus* **centroid size** **does** significantly vary across different precipitation regimes, when tested using Pairwise T-Tests with correction for multiple testing (pairwise.t.test function in stats package). Specimens from environments that are drier than modern levels are significantly larger than specimens from environments that are either equal to modern levels of precipitation or wetter.

**Supplementary Table 2: *E. fuscus* centroid size by precipitation**

| p-values | **Dry** | **Modern** |
| --- | --- | --- |
| **Modern** | **0.029** | - |
| **Wet** | **0.029** | 0.393 |

**✔** *E. fuscus* **centroid size** **does** significantly vary across different climatic regimes, when tested using Pairwise T-Tests with correction for multiple testing (pairwise.t.test function in stats package). Specimens from environments that are cooler and drier than modern day levels are significantly larger than specimens from both environments that are cooler and wetter than modern day, as well as environments that are warmer and with modern levels of precipitation.

The directionality of the difference is given by comparing **row** by **column**. E.g. **Cool, Wet** specimens are significantly **smaller** than **Cool, Dry** specimens.

**Supplementary Table 3: *E. fuscus* centroid size by climate**

| p-values | **Cool, Dry** | **Cool, Wet** |
| --- | --- | --- |
| **Cool, Wet** | **0.029**  **(smaller)** | - |
| **Warm,**  **Modern**  **Precip.** | **0.029**  **(smaller)** | 0.393 |

Shape - Climate

**✔** *E. fuscus* **shape** **does** significantly vary across different temperature regimes, when tested using Permutation Testing For Group Differences with correction for multiple testing (not applicable in this specific instance in any case; permudist function in Morpho package).

**Supplementary Table 4: *E. fuscus* shape by temperature**

| p-values | **Modern** |
| --- | --- |
| **Cool** | **0.0281** |

**X** *E. fuscus* **shape** **does not** significantly vary across different precipitation regimes, when tested using Permutation Testing For Group Differences with correction for multiple testing (permudist function in Morpho package).

**Supplementary Table 5: *E. fuscus* shape by precipitation**

| p-values | **Dry** | **Modern** |
| --- | --- | --- |
| **Modern** | 0.1257 | - |
| **Wet** | 0.8010 | 0.1257 |

**X** *E. fuscus* **shape**  **does not** significantly vary across different climatic regimes, when tested using Permutation Testing For Group Differences with correction for multiple testing (permudist function in Morpho package).

**Supplementary Table 6: *E. fuscus* shape by climate**

| p-values | **Cool, Dry** | **Cool, Wet** |
| --- | --- | --- |
| **Cool, Wet** | 0.8116 | - |
| **Warm,**  **Modern**  **Precip.** | 0.1158 | 0.1158 |

Centroid Size - Age

Each specimen is associated with an **Upper** and **Lower Age Estimate**. Using a Linear Model (lm function in stats package):

**✔** *E. fuscus* **Lower Age Estimate** **does** significantly predict **centroid size**. **p=0.0199**

**Older** specimens have a **larger** Centroid Size.

**✔** *E. fuscus* **Upper Age Estimate** **does** significantly predict **centroid size**. **p=0.00724**

**Older** specimens have a **larger** Centroid Size.

PCA

Scree Plots

**Supplementary Figure 1: Scree plot of PCA**

**Supplementary Table 13: Variation described per Principal Component**

|  | **PC1** | **PC2** | **PC3** | **PC4** | **PC5** | **PC6** |
| --- | --- | --- | --- | --- | --- | --- |
| ***fuscus*** | 36.70% | 16.34% | 13.26% | 7.25% | 5.94% | 4.64% |
| ***velifer*** | 20.89% | 17.66% | 11.87% | 7.83% | 6.35% | 5.83% |
|  | **PC7** | **PC8** | **PC9** | **PC10** | **PC11** | **PC12** |
| ***fuscus*** | 2.71% | 2.21% | 1.93% | 1.54% | 1.06% | 0.86% |
| ***velifer*** | 4.74% | 3.43% | 2.92% | 2.23% | 1.70% | 1.66% |
|  | **PC13** | **PC14** | **PC15** | **PC16** | **PC17** | **PC18** |
| ***fuscus*** | 0.70% | 0.67% | 0.57% | 0.45% | 0.37% | 0.31% |
| ***velifer*** | 1.51% | 1.18% | 1.13% | 0.87% | 0.82% | 0.74% |

*M. velifer* PC tests

PCs – Age

Each specimen is associated with an **Upper** and **Lower Age Estimate**. Using a Linear Model (lm function in stats package):

**✔** *M. velifer* **PCs 1 and 2 are** significantly predicted by both **Lower** and **Upper Age Estimates**.

**X** *M. velifer* **PC 3 is not** significantly predicted by either **Lower** or **Upper Age Estimates**.

**Supplementary Table 14: *M. velifer* PCs by age**

| p-values | **Lower Age Estimate** | **Upper Age Estimate** |
| --- | --- | --- |
| **PC1** | **0.00374** | **0.00521** |
| **PC2** | **0.00683** | **0.00507** |
| **PC3** | 0.125 | 0.0809 |

PCs – Climate

**X** *M. velifer* **PCs 1, 2 and 3 do not** significantly vary across different temperature regimes, when tested using Permutation Testing For Group Differences with correction for multiple testing (permudist function in Morpho package).

**Supplementary Figure 2: *M. velifer* PCs by temperature**


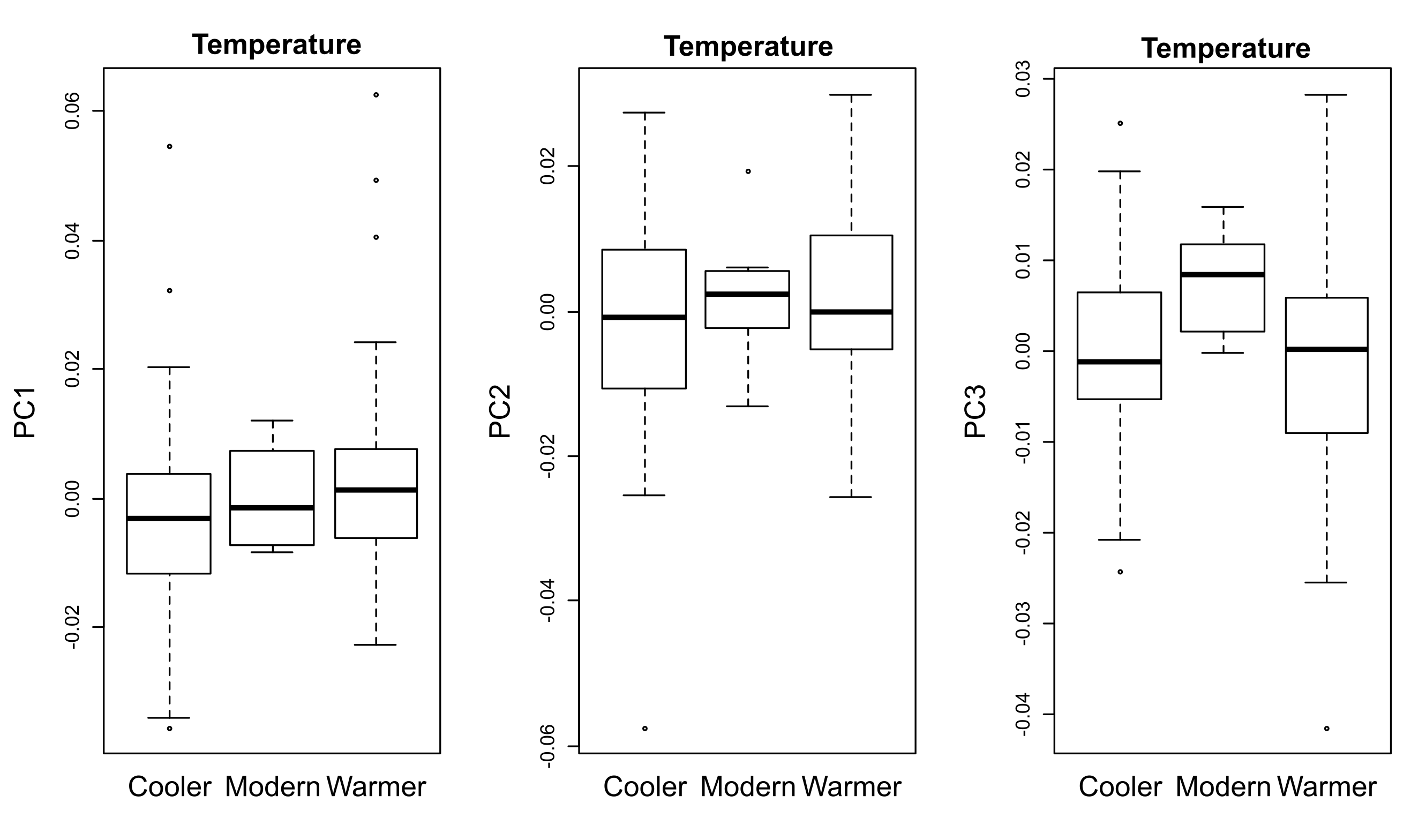


**Supplementary Table 15: *M. velifer* PC1 by temperature**

| p-values | **Cool** | **Modern** |
| --- | --- | --- |
| **Modern** | 1.0000 | - |
| **Warm** | 0.0564 | 1.0000 |

**Supplementary Table 16: *M. velifer* PC2 by temperature**

| p-values | **Cool** | **Modern** |
| --- | --- | --- |
| **Modern** | 0.9270 | - |
| **Warm** | 0.5058 | 0.9270 |

**Supplementary Table 17: *M. velifer* PC3 by temperature**

| p-values | **Cool** | **Modern** |
| --- | --- | --- |
| **Modern** | 0.2073 | - |
| **Warm** | 0.9261 | 0.2073 |

**X** *M. velifer* **PCs 1, 2 and 3 do not** significantly vary across different precipitation regimes, when tested using Permutation Testing For Group Differences with correction for multiple testing (permudist function in Morpho package).

**Supplementary Figure 3: *M. velifer* PCs by precipitation**


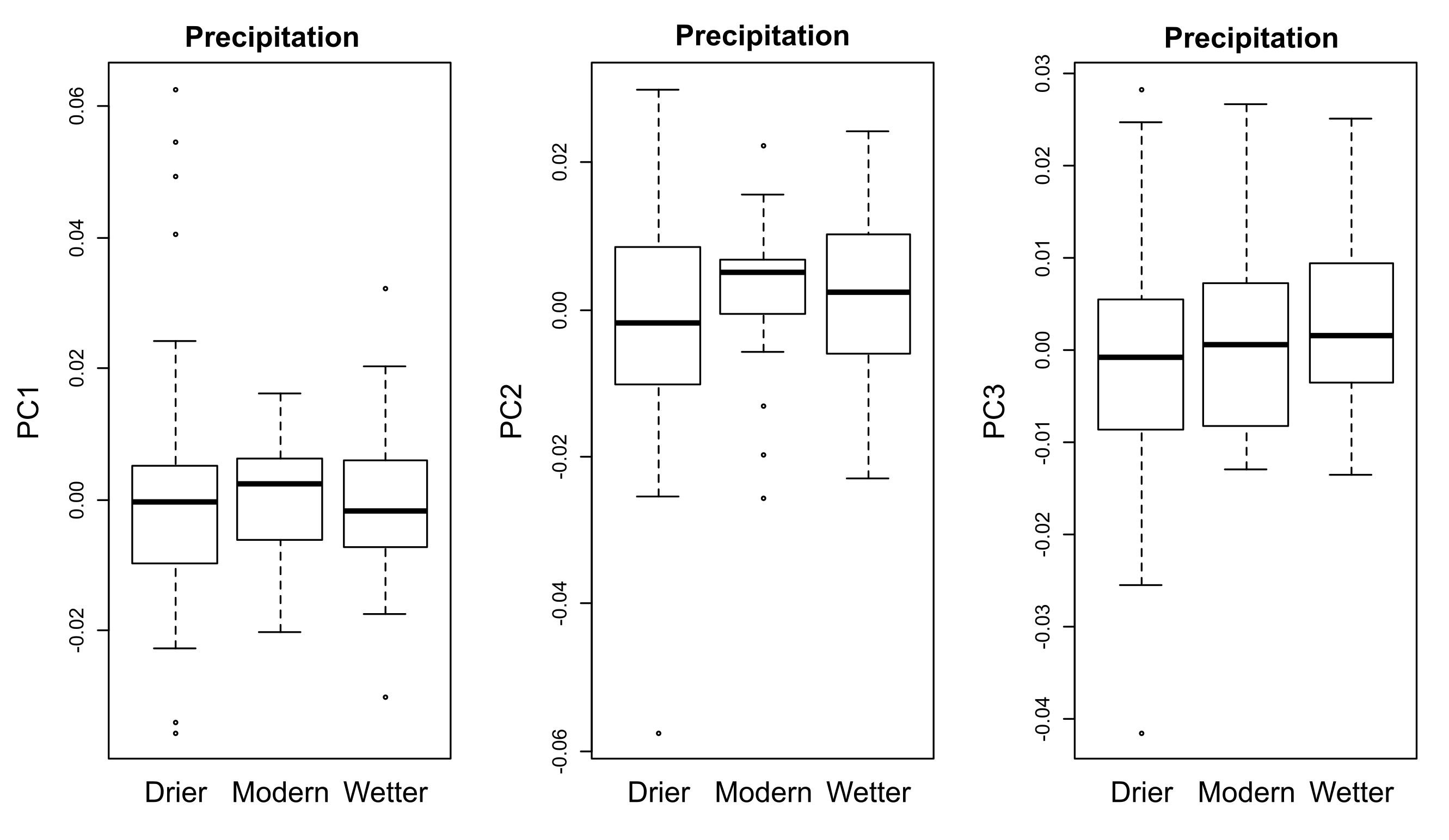


**Supplementary Table 18: *M. velifer* PC1 by precipitation**

| p-values | **Dry** | **Modern** |
| --- | --- | --- |
| **Modern** | 1 | - |
| **Wet** | 1 | 1 |

**Supplementary Table 19: *M. velifer* PC2 by precipitation**

| p-values | **Dry** | **Modern** |
| --- | --- | --- |
| **Modern** | 0.7546 | - |
| **Wet** | 0.2817 | 0.7546 |

**Supplementary Table 20: *M. velifer* PC3 by precipitation**

| p-values | **Dry** | **Modern** |
| --- | --- | --- |
| **Modern** | 0.7552 | - |
| **Wet** | 0.1218 | 0.7552 |

**✔** *M. velifer* **PCs 1, 2 and 3 do** significantly vary across different climate regimes, when tested using Permutation Testing For Group Differences with correction for multiple testing (permudist function in Morpho package). The sole significant relationship established is that between PC1 and the pairwise comparison of specimens from environments that are on the one hand cooler and drier than modern day and the on the other hand warmer and drier than modern day.

**Supplementary Figure 4: *M. velifer* PCs by climate**


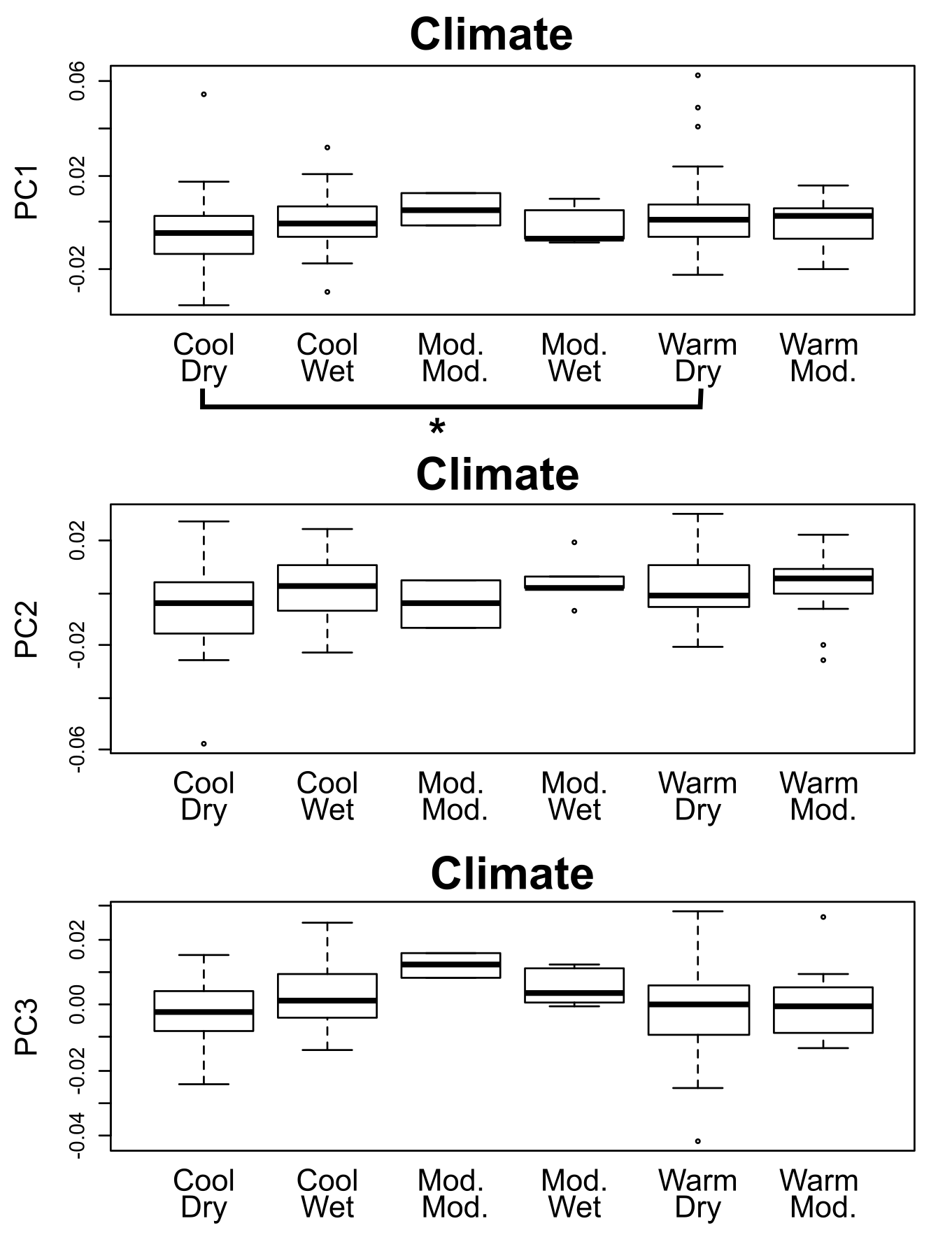


**Supplementary Table 21: *M. velifer* PC1 by climate**

| p-values | **Cool, Dry** | **Cool, Wet** | **Modern Temp., Modern Precip.** | **Modern Temp., Wet** | **Warm, Dry** |
| --- | --- | --- | --- | --- | --- |
| **Cool, Wet** | 1.0000 | - | - | - | - |
| **Modern Temp., Modern Precip.** | 1.0000 | 1.0000 | - | - | - |
| **Modern Temp., Wet** | 1.0000 | 1.0000 | 1.0000 | - | - |
| **Warm, Dry** | **0.0465** | 1.0000 | 1.0000 | 1.0000 | - |
| **Warm, Modern. Precip.** | 1.0000 | 1.0000 | 1.0000 | 1.0000 | 1.0000 |

**Supplementary Table 22: *M. velifer* PC2 by climate**

| p-values | **Cool, Dry** | **Cool, Wet** | **Modern Temp., Modern Precip.** | **Modern Temp., Wet** | **Warm, Dry** |
| --- | --- | --- | --- | --- | --- |
| **Cool, Wet** | 0.1815 | - | - | - | - |
| **Modern Temp., Modern Precip.** | 1.0000 | 1.0000 | - | - | - |
| **Modern Temp., Wet** | 1.0000 | 1.0000 | 1.0000 | - | - |
| **Warm, Dry** | 0.2716 | 1.0000 | 1.0000 | 1.0000 | - |
| **Warm, Modern. Precip.** | 0.6461 | 1.0000 | 1.0000 | 1.0000 | 1.0000 |

**Supplementary Table 23: *M. velifer* PC3 by climate**

| p-values | **Cool, Dry** | **Cool, Wet** | **Modern Temp., Modern Precip.** | **Modern Temp., Wet** | **Warm, Dry** |
| --- | --- | --- | --- | --- | --- |
| **Cool, Wet** | 0.7350 | - | - | - | - |
| **Modern Temp., Modern Precip.** | 0.8946 | 1.0000 | - | - | - |
| **Modern Temp., Wet** | 1.0000 | 1.0000 | 1.0000 | - | - |
| **Warm, Dry** | 1.0000 | 1.0000 | 1.0000 | 1.0000 | - |
| **Warm, Modern. Precip.** | 1.0000 | 1.0000 | 1.0000 | 1.0000 | 1.0000 |

PCs – Centroid Size

**X** *M. velifer* **PCs 1, 2 and 3 are not** significantly predicted by centroid size, when tested with a Linear Model (lm function in stats package).

**Supplementary Table 24: *M. velifer* PCs by centroid size**

| p-values | **Centroid Size** |
| --- | --- |
| **PC1** | 0.308 |
| **PC2** | 0.165 |
| **PC3** | 0.907 |

*E. fuscus* PC tests

PCs – Age

Each specimen is associated with an **Upper** and **Lower Age Estimate**. Using a Linear Model (lm function in stats package):

**X** *E. fuscus* **PCs 1, 2 and 3 were not** significantly predicted by either **Lower** or **Upper Age Estimates**.

**Supplementary Table 25: *E. fuscus* PCs by age**

| p-values | **Lower Age Estimate** | **Upper Age Estimate** |
| --- | --- | --- |
| **PC1** | 0.364 | 0.322 |
| **PC2** | 0.366 | 0.409 |
| **PC3** | 0.940 | 0.966 |

PCs – Climate

**X** *E. fuscus* **PCs 1, 2 and 3 did not** significantly vary across different temperature regimes, when tested using Permutation Testing For Group Differences with correction for multiple testing (permudist function in Morpho package).

**Supplementary Figure 5: *E. fuscus* PCs by temperature**


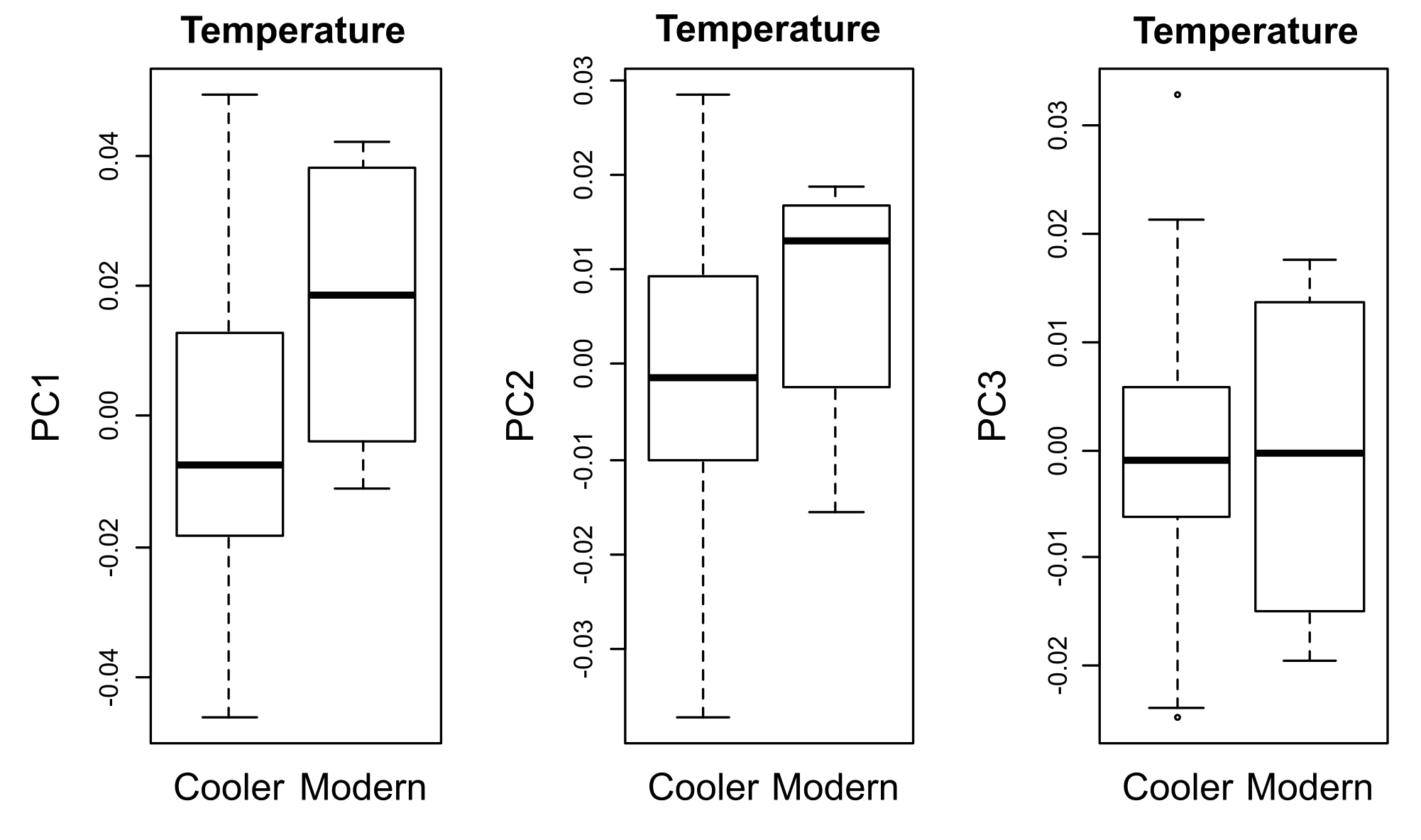


**Supplementary Table 26: *E. fuscus* PC1 by temperature**

| p-values | **Modern** |
| --- | --- |
| **Cool** | 0.1828 |

**Supplementary Table 27: *E. fuscus* PC2 by temperature**

| p-values | **Modern** |
| --- | --- |
| **Cool** | 0.325 |

**Supplementary Table 28: *E. fuscus* PC3 by temperature**

| p-values | **Modern** |
| --- | --- |
| **Cool** | 0.9184 |

PCs – Centroid Size

**X** *M. velifer* **PCs 1, 2 and 3 are not** significantly predicted by centroid size, when tested with a Linear Model (lm function in stats package).

**Supplementary Table 29: *E. fuscus* PCs by centroid size**

| p-values | **Centroid Size** |
| --- | --- |
| **PC1** | 0.370 |
| **PC2** | 0.554 |
| **PC3** | 0.833 |
